# Towards One Health clinical management of zoonoses: A parallel survey of Australian general medical practitioners and veterinarians

**DOI:** 10.1111/zph.12799

**Published:** 2020-12-31

**Authors:** Sandra G. Steele, Robert Booy, Ramesh Manocha, Siobhan M. Mor, Jenny‐Ann L. M. L. Toribio

**Affiliations:** ^1^ Faculty of Science Sydney School of Veterinary Science The University of Sydney Sydney NSW Australia; ^2^ National Centre for Immunisation Research and Surveillance Children's Hospital at Westmead Sydney NSW Australia; ^3^ Faculty of Medicine and Health School of Medicine The University of Sydney Sydney NSW Australia; ^4^ Healthed Burwood NSW Australia; ^5^ Institute of Infection, Veterinary and Ecological Sciences University of Liverpool Merseyside UK

**Keywords:** general practitioners, One Health, veterinarians, zoonoses

## Abstract

General medical practitioners (GPs) and veterinarians have different but complementary knowledge and skills, with potential to enhance clinical management of zoonoses in human and animal patients through taking a One Health approach that promotes cross‐professional collaboration. Ability and willingness to engage within this framework is contingent on knowledge of endemic zoonoses and an understanding of the diversity of professional roles; however, previous research suggests that this is lacking. A unique parallel survey of Australian GPs and veterinarians was implemented to ascertain clinician experience, concern, confidence and current practices regarding zoonoses management as well as willingness to engage in cross‐professional collaboration where it is beneficial to overall health outcomes. Responses from 528 GPs and 605 veterinarians were analysed. Veterinarians in clinical practice were found to more frequently diagnose zoonoses; have greater concern about zoonoses; be more confident in diagnosing, managing and giving advice about the prevention of zoonoses; more likely to give advice about managing the risk of zoonoses; and more likely to initiate cross‐professional referral compared to GPs (*p* < .001 in all areas, adjusted for other factors). The findings of this study indicate a need for change in both clinical and continuing professional education, especially for GPs, in order to better equip them in the area of zoonoses management. Exploration of pathways to encourage and facilitate cross‐professional referral and collaboration will further improve clinical outcomes for both human and animal patients.

**Impacts**

- This study is a unique parallel survey of Australian general medical practitioners (GPs) and veterinarians.
- Compared to GPs, veterinarians were found to more frequently diagnose zoonoses; have greater concern about zoonoses; be more confident in diagnosing, managing and giving advice about the prevention of zoonoses; more likely to give advice about managing the risk of zoonoses; and more likely to initiate cross‐professional referral.
- Addressing the significant differences between practitioner groups will require targeted changes in both medical and veterinary education as well as continuing practitioner education in the area of zoonoses.

## 1 INTRODUCTION

Domestic animals occupy an increasingly significant place in Australian life with two thirds of households owning an estimated 29 million pets (Animal Medicines Australia, 2019) and a further 92 million livestock animals and 137 million chickens raised on farms (Australian Bureau of Statistics, 2019). Many companion animals are considered integral members of the family, sharing the house, food and bed with their owners (Animal Medicines Australia, 2019; Chomel & Sun, 2011). Such close contact creates opportunities for disease transmission between animals and humans (zoonoses). In the last decade, researchers have sounded alarm about the changing pattern of zoonoses, both in Australia (Wang, 2011) and globally (Jones et al., 2008). Deforestation and increased urbanization have increased the interface between wildlife, domestic animals and human populations, giving rise to novel zoonotic diseases such as Hendra virus (Mahalingam et al., 2012) and Australian Bat Lyssavirus (ABLV) (Annand & Reid, 2014), which have caused fatal infections in both animals and humans in Australia. In addition, the epidemiology of some well‐known zoonotic diseases is changing. For example, Q fever, which is the most common direct zoonoses in Australia (565 cases reported in 2019; (Department of Health, 2019b,), was predominantly recognized as a disease affecting occupationally exposed groups; now, it is being increasingly identified in people without known risk factors (Clutterbuck et al., 2018; Eastwood et al., 2018). Such shifting disease patterns require an adaptive workforce that is capable of detecting and managing zoonoses in both human and animal patients (Rabinowitz & Conti, 2010).

General medical practitioners (GPs) and veterinarians play a critical role in front line health care and disease surveillance for humans and animals (Rabinowitz & Conti, 2013; Shomaker et al., 2013; Steele et al., 2018). While there are obvious differences in patient cohorts and species‐specific disease presentations, both professions share the common goal of optimizing health outcomes for their respective patients. Given the different but complementary knowledge and skills of each practitioner group, there is potential to enhance clinical management of zoonoses through taking a One Health approach that promotes cross‐professional collaboration (Eussen et al., 2017; Grant & Olsen, s in much of the world, there is no formal referral pathway^1999^; Kahn et al., 2007; Rabinowitz & Conti, 2010; Steele et al., 2018).

Australia has a long‐established universal healthcare scheme (Medicare) wherein GPs provide primary care services with established referral pathways to medical specialists (Department of Health, 2019a,). As in much of the world, there is no formal referral pathway between medical practitioners and veterinarians (Speare et al., 2015); however, some relationships exist between veterinarians and medical professionals in academia and government as well as informally between some GPs and veterinary practitioners. Australia is currently the only Organisation for Economic Co‐operation and Development (OECD) country without a national multidisciplinary body focused on disease prevention, investigation and control (Australian Medical Association, 2017), and therefore lacks a coordinated, government‐supported structure promoting cross‐professional infectious disease management at both a governance and clinician level.

The current lack of effective One Health practice at the clinician level stems from varying knowledge of common endemic zoonoses and an understanding of the One Health paradigm amongst veterinarians and GPs (Eussen et al., 2017; Grant & Olsen, 1999; Hodgson et al., 2019; von Matthiessen et al., 2003; Rabinowitz & Conti, 2013). Previous research conducted by us and other researchers has suggested that the genesis of this problem lies in a number of factors, including the limited coverage of these subjects in clinical degree programs as well as continuing professional education (Hodgson et al., 2019; Kahn et al., 2008; Marcotty et al., 2013; Steele et al., 2018, 2019; Togami et al., 2018). Further, the structure of medical and veterinary services and existence of professional silos results in clinicians tending to act individually rather than collaboratively and cooperatively (Rabinowitz & Conti, 2010; Speare et al., 2015). GPs and veterinarians may also have differing perceptions of the importance and risks of different zoonotic diseases as well as varying levels of knowledge and clinical experience, stemming from differences in the clinical severity in and transmissibility of zoonotic disease from in their respective patient populations. Such differences are likely to influence clinical practices with regard to zoonoses management.

Worldwide, there are no studies comparing medical practitioners and veterinarians with regard to general zoonotic disease management. A small number of studies have focused exclusively on management of immunosuppressed patients (Grant & Olsen, 1999; Hill et al., 2012; von Matthiessen et al., 2003) or a specific zoonoses (Hennenfent et al., 2018). In order to address this gap, we conducted a parallel survey of GPs and veterinarians guided by priority areas established in our previous research and current literature. Specifically, the aims of the surveys were to determine clinician: (1) experience diagnosing zoonoses in their practice; (2) concern about zoonoses; (3) confidence in diagnosing, treating and managing zoonoses; and (4) current practices and willingness to engage in cross‐professional collaboration where it is beneficial to overall health outcomes.

## 2 METHODS

### 2.1 Study design

A cross‐sectional study using two parallel surveys directed at Australian GPs and veterinarians in clinical practice was conducted informed by research priorities established by the researchers in previous studies (Steele et al., 2018, 2019). Survey questions addressed participant experience, concern, confidence and practices regarding zoonotic diseases using binary response (yes/no), five‐point Likert‐scale and open‐ended questions to further explore clinician responses (See Appendix 1). The respective questionnaires were identical apart from wording appropriate for each practitioner group to enable statistical comparisons, and the inclusion of some questions addressing areas unique to each practitioner group. Demographic data including year and place of graduation, postcode of current workplace and previous professional experience in rural areas or developing countries were also collected. In addition, veterinarians were asked which type of practice they were involved in (e.g. small animal practice). A sample size of 380 for GPs and 372 for veterinarians was calculated based on numbers of practitioners (AHPRA, 2019; Australian Veterinary Boards Council, 2019) to give a 95% level of confidence and 5% margin of error.

The survey questionnaires were implemented using the secure, web‐based application, REDCap, hosted at the University of Sydney. The survey links were distributed through a number of forums including practitioner education groups, professional associations, practitioner boards and the researchers’ networks via email, newsletters, social media and professional conferences. A summary of modes of recruitment is provided in Table S1. The GP survey was conducted between March and May 2019, and the veterinary survey between May and July 2019. The respective questionnaires were pre‐tested with a small number of medical practitioners, and veterinarians before distribution to ensure the wording of questions were unambiguous to practitioners from both groups.

### 2.2 Data analysis

#### 2.2.1 Quantitative data

Survey data from REDCap were downloaded into Microsoft^®^ Excel 2016 v16.31. Responses were received from 714 GPs and 856 veterinarians, of which 437 were excluded from analysis. Reasons for exclusion included: only demographic data provided (*N* = 163 GPs, *N* = 162 veterinarians); participant was not a GP (*N* = 12, including nurse practitioners, paediatrician, hospital‐based practitioners) or veterinarian in clinical practice (*N* = 65, including government veterinarians, industry veterinarians and academics); or less than one third of the survey was completed (*N* = 11 GPs, *N* = 24 veterinarians). All remaining surveys were included in the analysis.

Descriptive analysis was performed in IBM SPSS Statistics, Version 26 (SPSS Inc, Chicago, US). Frequency tables of demographics were compiled, and differences between practitioner groups assessed using chi‐squared or Mann–Whitney U tests for categorical and ordinal variables, respectively. Place of graduation (open‐ended question) was re‐coded into a categorical variable denoting world region. Similarly, place of work was re‐coded into a categorical variable (urban or rural/semi‐rural area) based on postcode, using data from the Australian Bureau of Statistics Remoteness Structure (Australian Bureau of Statistics, 2018). Year of graduation was aggregated into 10‐year groups (2010–2019, 2000–2009, 1990–1999 and 1989 and before). For veterinary practitioners, data from those who selected ‘other’ for practice type was differentiated and coded appropriately. Finally, Likert‐scale data and binary data from outcomes of interest were plotted using diverging stacked bar graphs, a recommended method for representation of these rating scales (Heiberger & Robbins, 2014; Heiberger & Heiberger, 2017) using the HH‐package (Heiberger & Heiberger, 2017) in R, Version 3.6.3 (R Core Team, Vienna, Austria).

Logistic regression modelling was performed in R to investigate the difference between GPs and veterinarians in areas of interest, namely practitioner experience as determined by diagnosis of zoonoses (binary outcome), concern (ordinal outcome), confidence and practices related to zoonoses. Models examining practitioner confidence were developed around 3 areas, namely self‐rated confidence in diagnosing common zoonoses, managing common zoonoses and providing advice to patients/clients about prevention of common zoonoses (ordinal outcome). Models examining practices focussed on frequency of discussing zoonotic risk with patients/clients (ordinal outcome) and whether practitioners had ever made a cross‐professional referral (binary outcome).

For ordinal outcomes, five‐point Likert‐scale data were collapsed into 3 outcomes (1 + 2, 3, and 4 + 5) and regression analysis performed using a cumulative link model. The main explanatory variable of interest was a binary variable denoting GP/veterinarian status. Place of work (rural versus urban), year of graduation (proxy for age/clinical experience), gender, country of graduation (Australia or overseas) and whether practitioners had worked in a developing country were thought to be potential confounders; therefore, an a priori decision was made to include them in the regression models. Multicollinearity between explanatory variables was tested using the variance inflation factor (VIF) which was <2 for all comparisons. The assumption of proportionality was evaluated using the nominal test, which was not significant (*p* > .05) for all outcomes. Overall model fit was assessed using the likelihood‐ratio chi‐square test with the null hypothesis being rejected in all cases (*p* < .001).

For binary outcomes, logistic regression analysis was conducted using a generalized linear model using the same explanatory variables above. The Hosmer–Lemeshow goodness of fit test showed good model fit (*p* > .05). Odds ratios were calculated using GPs as the reference group (OR = 1), and 95% confidence intervals were determined. P values were determined using a Wald test.

A multivariable analysis using similar modelling was performed on data from veterinarians to investigate the difference in outcomes of interest between small animal practitioners and veterinarians in other areas of practice, namely mixed practice, production animal, equine, avian, exotics and wildlife.

### 2.3 Qualitative data

Responses to open‐ended questions were analysed using NVivo v 11.4.3 and manual methods. Lists of data were compiled, or, where appropriate, topic areas were determined using principles of thematic analysis by one researcher (SS) and reviewed by other team members. Participant quotations included in the text to illustrate topic areas are italicized and identified by professional grouping.

## 3 RESULTS

### 3.1 Demographics

Responses were analysed from 528 GPs and 605 veterinarians, representing approximately 2.0% (GPs) and 4.5% (veterinarians) of the national workforce (AHPRA, 2019; Australian Veterinary Boards Council, 2019). The demographic characteristics of participants are shown in Table 1. Differences existed between GPs and veterinarians with regard to gender (*p* = .007), professional experience as represented by year of graduation (*p* < .001), number of Australian graduates (*p* < .001), rural employment (*p* = .004) and prior experience working in a developing country (*p* < .001). All GP participants identified as being general medical practitioners with 2.1% also having additional roles. Three quarters (75.9%) had further qualifications with 286 being Fellows of the Royal Australian College of General Practice (FRACGP), a requirement for Australian GPs instituted in 1996 for vocational registration, with those working in general practice before this time being able to apply for grandfathering onto the register if they met eligibility requirements. In addition, 96 GPs had diplomas related to specific areas of general practice such as Obstetrics and Gynaecology, and Paediatrics. The veterinarians identified a broad range of practice types, with the largest percentage (65.6%) being small animal practitioners. More than half (54.4%) had other qualifications including Membership or Fellowship of the Australian and New Zealand College of Veterinary Scientists and vocationally related master's degrees.

**TABLE 1:** Demographics of Australian general medical practitioners (GPs; *n* = 528) and veterinarians (*n* = 605) who participated in the online surveys

Professional Group	GP *N* = 528		Vet *N* = 605		*p* Value^a^
	*N*	%	*N*	%	
Gender					
Male	170	32.2	151	25.0	.007^b^
Female	355	67.2	451	74.5	
Transgender	0	0	1	0.2	
Prefer not to Say	3	0.6	2	0.3	
University Degree					
Australia	390	73.9	543	89.7	<.001^c^
Europe	40	7.6	31	5.2	
Americas	4	0.8	8	1.3	
Africa	21	4.0	8	1.3	
Asia	41	7.8	1	0.2	
Oceania (not Australia)	21	4.0	12	2.0	
Unknown	11	2.1	2	0.3	
Year of Graduation					
<1989	244	46.2	138	22.8	<.001^d^
1990–1999	112	21.2	116	19.2	
2000–2009	114	21.6	144	23.8	
2010–2019	58	11.0	205	33.9	
Unknown	0	0	2	0.3	
State or territory					
Australian Capital Territory	9	1.7	31	5.1	N/A
New South Wales	166	31.4	171	28.3	
Northern Territory	9	1.7	20	3.3	
Queensland	131	24.8	97	16.0	
South Australia	38	7.2	38	6.3	
Tasmania	8	1.5	33	5.5	
Victoria	117	22.2	161	26.6	
Western Australia	50	9.5	54	8.9	
Location					
Urban	335	63.4%	333	55.0%	.004
Rural and Semi‐Rural	193	36.6%	272	45.0%	
Extra qualifications	401	75.9%	276	45.6%	<.001^e^
Have you worked in:					
A rural or remote area					
Yes	362	68%	406	67.1%	.601
No	166	31.4%	199	32.9%	
A developing country					
Yes	166	31.4%	106	17.6%	<.001
No	362	68.6%	497	82.4%	
Practice Type (veterinarian)					
**S**mall animal			396	65.6%	
Mixed practice			138	22.9%	
Equine			20	3.3%	
Production animals			19	3.2%	
Exotics			13	2.2%	
Avian			7	1.2%	
Other			4	0.7%	

^a^ Chi‐squared test.

^b^ Male v female.

^c^ Australian graduates v overseas graduates.

^d^ Mann–Whitney *U* test.

^e^ Pathways to extra qualifications are different between professions, so may not be equivalent across groups.

### 3.2 Descriptive and qualitative analyses

**FIGURE 1:**
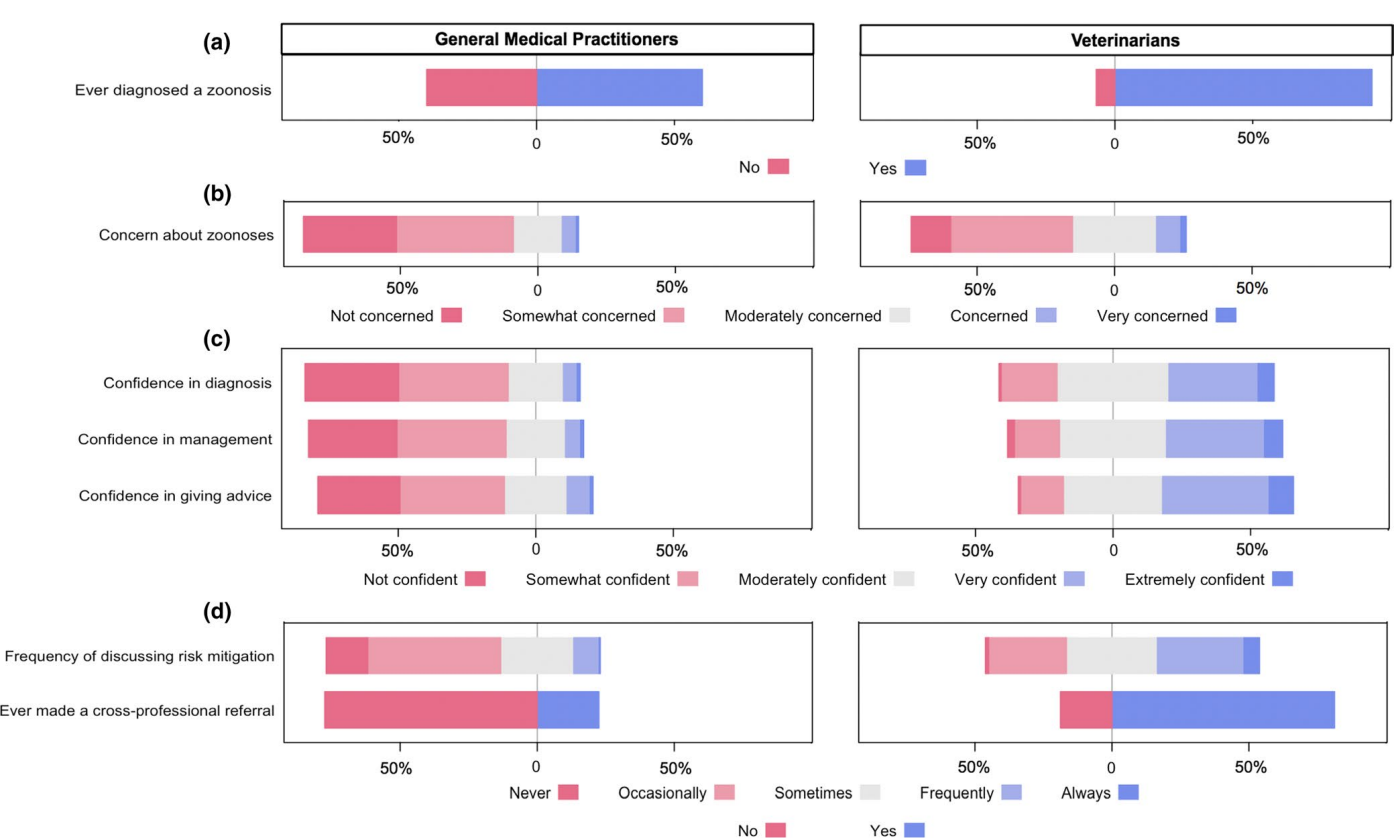
Responses by Australian general medical practitioners (GPs; *n* = 528) and veterinarians (*n* = 605) regarding clinician experience (Panel a), concern (Panel b), confidence (Panel c) and practices (panel d) related to zoonoses [Colour figure can be viewed at wileyonlinelibrary.com]

Figure 1 shows the responses from GPs and veterinarians with regard to experience, concern, confidence and practices related to zoonoses. Bivariate comparisons between GPs and veterinarians are shown in Table S2. Veterinarians reported being more concerned about zoonoses (*p* < .001) as well as more confident in the diagnoses (*p* < .001), management (*p* < .001), and giving advice about the prevention of zoonoses (*p* < .001).

#### 3.2.1 Practitioner experience with zoonoses

Approximately 60% of GPs and 93% of veterinarians reported that they had diagnosed a zoonotic disease in a patient (GP 60.1% *N* = 316, Vet 93.2% *N* = 564 *p* < .001; Figure 1 and Table S2). Clinicians who had diagnosed a zoonosis were asked to identify which zoonotic diseases they had diagnosed in the practice they were currently working in. GPs named 63 different diseases/agents with the most frequently diagnosed being bacterial gastroenteritis (148/316;46.8%), Q fever (36.1%), dermatophytosis (22.2%), giardiasis (17.1%) and leptospirosis (16.1%). Veterinarians named 84 diseases/agents, including a number of rarely encountered zoonoses. The most commonly diagnosed were bacterial gastroenteritis (488/564; 86.5%), dermatophytosis (77.8%), toxoplasmosis (26.4%), common endoparasites of companion animals (round, hook and tapeworm; 22.3%) and sarcoptic mange (20.0%). A list of the 20 most commonly diagnosed diseases and agents can be found in Table S3.

Participants were asked if they had noticed any changes in the patterns of zoonotic disease occurrence in their region that could be associated with environmental changes. Both GPs (70/528) and veterinarians (76/605) who responded in the affirmative recognized more frequent presentations of zoonotic diseases, some of which were previously unrecognized in their area. GPs reported cases of Q fever in patients from ‘non‐traditional occupations’ or ‘no animal exposure’, attributing this to spread by dust due to dryer conditions. Others noted an increase in vector‐borne diseases such as Ross River fever attributed to changes in rainfall patterns or differences associated with ‘hav[ing] become much more suburban and less rural’. Veterinarians were markedly concerned about the recent occurrence of Hendra virus further south in New South Wales than previously reported, linking this with disturbance of flying fox populations with ‘stressors due to habitat loss increasing pathogen loads in wildlife patients’. Instances of illness and death in wild birds and other atypical disease occurrences were specifically noted such as recent cases of canine leptospirosis in Sydney, changing range of *Brucella suis* cases, increased reports of salmonellosis in a number of species and unusual spikes of endoparasitism such as liver flukes in ruminants and Angiostrongylosis in dogs.

#### 3.2.2 Practitioner concern about zoonotic diseases

GPs were less likely than veterinarians to express any level of concern about zoonoses (GPs 66.2% *N* = 349, Vet 85.6% *N* = 518, *p* < .001; Figure 1 and Table S2). Practitioners who expressed some level of concern were asked to list which zoonotic diseases concerned them the most in their current workplace. GPs listed 74 diseases/agents with Q fever (174/349; 55%), bacterial gastrointestinal diseases (24%), rabies (15%), toxoplasmosis (14%) and leptospirosis (13%) being of greatest concern. Veterinarians listed 68 diseases/agents with dermatophytosis (232/518; 45%), bacterial gastrointestinal diseases (42%), Q fever (28%), psittacosis (21%) and toxoplasmosis (19%) being of greatest concern. Some veterinarians showed disquiet about the potential of both undiscovered zoonoses and the increasing risk of antimicrobial‐resistant bacteria, with one participant commenting that ‘*the unknown is my biggest worry’*. A list of the top 20 diseases of concern for GPs and veterinarians is shown in Table S4.

#### 3.2.3 Practitioner practices regarding zoonotic diseases

##### Discussion of risk of zoonotic diseases

Approximately 10% of GPs and 37% of veterinarians stated that they frequently or always discussed potential risk of zoonotic diseases or strategies to prevent these with their patients/clients (GPs 9.7% *N* = 51, Vet 37.2% *N* = 225, *p* < .001; Figure 1 and Table S2).

Veterinarians described risk mitigation as a professional responsibility and stated that they routinely incorporated it into wellness consultations, consultations with new clients or new pets and as part of health management programs for production animals. One veterinarian commented that ‘I feel that vets should make owners aware of potential disease risks from animals because it seems they don't get this information given to them elsewhere’. Veterinarians recounted routinely collecting information to ascertain the risk of zoonoses to their clients and family in order to direct recommendations regarding risk mitigation. This included in‐contact people (to determine whether there are immunocompromised family members); diet of companion animals with ‘raw feeding’ singled out as a significant risk factor; specific animal clinical presentations (mainly diarrhoea and reproductive complications, especially abortions); animal species perceived to have a higher risk of zoonotic disease transmission (birds, exotic pets, goats and sheep) and when animals appeared poorly cared for. Information regarding hand and food hygiene practices were seen as an essential component of risk management advice.

GPs most frequently discussed risk mitigation with their patients as part of routine travel advice, especially to overseas and rural and remote Australia, with specific focus on rabies and vector‐borne diseases. Discussion of risk was prompted by patient occupation (predominantly those in animal‐related industries such as farmers, veterinarians and veterinary nurses, wildlife carers, abattoir workers), involvement in recreational activities such as pig hunting and water sports, recurrent gastrointestinal illness and pregnancy. Some GPs recognized factors related to their local region which significantly increased patient risk of infection, one remarking they ‘discuss [risk factors] and record on most patients’ charts. Important in this community ‐ if not directly employed in Agriculture Industries, social exposure puts them in the middle of risk environment. Simply ‐ everybody in this town is at risk…’

GPs were also asked about the clinical situations (if any) that would prompt them to ask about animal contact. Potential ‘red flags’ that GPs reported may prompt questioning were as follows: unusual clinical signs or diagnostic uncertainty, often accompanied by a history of travel; exclusion of more likely causes of the patient's symptoms; pyrexia of unknown origin/recurrent fevers, recurrent gastrointestinal symptoms, skin lesions, arthralgia, respiratory signs, malaise and ‘flu like illnesses’. Although bacterial gastrointestinal diseases were stated as amongst the zoonoses of greatest concern to GPs, only 37.9% of GPs reported asking about animal contact in the context of patients with diarrhoea and/or vomiting.

##### Referral practices

Fewer GPs reported recommending cross‐professional referral when presented with a patient with or at risk of a zoonoses compared with veterinarians (GP 22.5% *N* = 118, Vet 81.2% *N* = 491, *p* < .001; Figure 1 and Table S2). Nonetheless, 67.7% of GPs said they would consider referral of patients to a veterinarian who has had extra training in zoonotic diseases in order to reduce the risk of zoonotic infection in their patients who are at risk.

For those that have practised cross‐professional referral, 78.8% of GPs (93/118) and 91.2% of veterinarians (448/491, 91.2%) gave patients/clients a verbal recommendation only. GPs more frequently provided written referrals (9.4%) or made phone calls (3.4%) than veterinarians (0.6% and 0.6%, respectively).

When asked about specific reasons for cross‐professional referral, responses from GPs and veterinarians fell into two major topics: (1) diagnosis or suspicion of a zoonoses in a patient; and (2) mitigation of risk for patient/client at higher risk of zoonotic exposure or infection. Veterinarians also described referral to GPs in specific circumstances such as exposure to bats due to the risk of Australian Bat Lyssavirus (ABLV) and animal bite injuries. Quotations from participants illustrative of these situations are presented in Table 2.

**TABLE 2:** Reasons for cross‐professional referral for zoonoses management, as stated by Australian general medical practitioners (GPs) and veterinarians who participated in the online surveys

Reasons for referral	Examples of reasons for referral
GPs	
Diagnosis or suspicion of a zoonoses in a patient.	
Specific scenarios	‘… multiple family members had similar rashes or coughs’ ‘campylobacter in patient whose dog had bad diarrhoea’ ‘Patient with atypical TB’ **‘**patient with Q fever; patient with salmonella and pet lizard also had salmonella’
Patient with skin lesions	‘Ringworm ‐ suggested they seek review of their cats/ kittens’ ‘The patient had got tinea from the dog.’
Exposure to ectoparasites/endoparasites	‘Anyone with worms I suggest that if they have a pet, they should make sure they are up to date with their worm prevention.’ ‘Usually people feel confident worming/treating their animals, rightly or wrongly’
Mitigation of patient risk	
Occupational exposures, concern about in‐contact animals.	‘To ensure their herds are screened and vaccinated as appropriate. Follow veterinary advice to adopt practices to prevent exposure.’ ‘Where the animals were not particularly well looked after’ ‘Farm workers, meat worker or animal keepers’ ‘Advice to promptly involve the vet in the care of acutely ill animals and not to self‐treat or use dubious alternative management.’
Pregnancy or history of recurrent miscarriage	‘Pregnant women (or planning) and cats’ ‘Cats & dogs, birds. Repetitive miscarriages’
Veterinarians	
Diagnosis or suspicion of a zoonoses in patient or client with typical clinical signs.	
Specific scenarios	‘Poultry flock diagnosed with aspergillosis and owner presenting with coughing, difficulty breathing etc’ ‘..diarrhoea which spread through a breeding dog facility. The humans began to have symptoms before our PCR returned so I encouraged all affected humans to consult their GP.’ ‘Client had symptoms of leptospirosis (directed to hospital)’
Client with skin lesions	‘Human with skin lesions that I suspected were ringworm’
Exposure to ectoparasites/endoparasites	‘Taenia saginata cysts reported in beef from a farm which had an overflowing septic tank.’ ‘Carer of a young baby who was treating calves with severe cryptosporidium.’
Mitigation of client risk	
Occupational exposures, concern about in contact animals	‘A friend of his had undiagnosed orchitis. He was a pig hunter. I recommended that he be tested for B. suis’ ‘Unvaccinated producers re. Q‐fever’ ‘MRSA cultured from a wound ‐ likely patient contracted from owner however owner had future surgery planned and so recommended discussing with their surgeon prior to surgery.’
Immunocompromised clients	‘Canine patient with XDR MRSA ‐ especially as owner's grandchild on chemo and dog lived inside the house’ ‘Owner was pregnant and had new kitten, recommend consult with GP to discuss toxoplasmosis and other zoonosis and make a risk‐management plan on the human side (made advice on minimising contact on the animal side, too)’ ‘Immunocompromised owner following liver transplant with dog which had recurrent GI infections including salmonella and giardia’
Direct contact with a bat	‘A man who was bitten by a bat which his dog was also exposed to’ ‘following confirmation that a bat which had been in contact with a client's dog was ABLV positive’
Animal bite injuries	‘Any owner that was ever bitten or badly scratched by their animal.’

Despite their reported knowledge of both human and animal manifestations of zoonoses, veterinarians perceived clear boundaries of practice, with one summarizing ‘I'm not going to advise [the client] on their health. I'll discuss the pet; they need to see their GP’. Another veterinarian added they are ‘not legally able to provide a diagnosis for a person’. Concerningly, veterinarians reported animals being presented for euthanasia on the basis of the advice given by a GP, for example, ‘GP recommended euthanasia for a newly acquired cat because child had skin lesion (no testing done on child to confirm cat's culpability)’ and more generally; ‘I have had doctors insist family pets be euthanased when humans are diagnosed with potential zoonoses without the animal concerned being examined on a number of occasions’.

Despite the high percentage of veterinarians (91.2%) that reported recommending cross‐professional referral, few GPs (6.1%) could recall having a patient sent to them by a veterinarian. Reasons described by GPs for a patient who had been advised to seek medical advice included potential exposure to a zoonotic disease such as ABLV or leptospirosis (6.1%), concern about risk factors for a zoonotic disease such as Q fever (4.8%) and following an animal‐related injury (17.6%).

### 3.3 Multivariable analysis

Results of linear and ordinal regression models comparing responses of GPs and veterinarians adjusted for other factors are shown in Table 3, with complete model output presented in Table S5. The sub‐analysis of veterinarian responses based on practitioner type is shown in Table S6.

**TABLE 3:** Results of multivariable analysis comparing responses of Australian general medical practitioners (GPs; *n* = 528) and veterinarians (*n* = 605) who participated in the online surveys

Experience with zoonotic diseases ‘Have you ever diagnosed a zoonotic disease in a patient?’						
	Yes	No		OR^a^, ^b^	95% CI	*p* Value^c^
GPs^d^	316 (60.1%)	210 (39.9%)		1		
Veterinarians	564 (93.2%)	41 (6.8%)		9.36	6.31–13.87	<.001

Concern about zoonoses ‘How concerned are you about zoonotic diseases in the practice you currently work in?’						
	Not/somewhat concerned	Moderately concerned	Very/extremely concerned			
GPs^e^	402 (76.3%)	94 (17.8%)	31 (5.9%)	1		
Veterinarians	355 (58.7%)	185 (30.6%)	65 (10.7%)	2.34	1.76–3.11	<0.001

Confidence regarding zoonoses ‘Overall, how confident do you feel in your ability to:’						
	Not/somewhat confident	Moderately confident	Confident/very confident			
**a: Diagnosis** **‘Diagnose common zoonotic diseases’**						
GPs^f^	386 (73.8%)	105 (20.1%)	32 (6.1%)	1		
Veterinarians^g^	127 (21.1%)	245 (40.7%)	230 (38.2%)	13.99	10.38–18.85	<.001
**b: Management** **‘Manage common zoonotic diseases’**						
GPs^f^	375 (71.7%)	113 (21.6%)	35 (6.6%)	1		
Veterinarians^g^	114 (18.9%)	234 (38.9%)	254 (42.2%)	15.03	11.14–20.29	<.001
**c: Advice** **‘Give advice about prevention of common zoonotic diseases’**						
GPs^f^	355 (67.9%)	119 (22.7%)	49 (9.4%)	1		
Veterinarians^g^	99 (16.4%)	217 (36.1%)	286 (47.5%)	12.98	9.71–17.36	<.001

Practices regarding zoonoses 3a: Zoonotic Risk ‘How often do you discuss potential risk of zoonotic diseases or strategies to prevent these with your patients/clients?’						
	Never/occasionally	Sometimes	Frequently/always			
GPs	336 (63.7%)	141 (26.7%)	51 (9.7%)	1		
Veterinarians	179 (29.6%)	201 (33.2%)	225 (37.2%)	4.95	3.82–6.42	<.001

3b: Referral ‘Have you ever recommended a patient with or at risk of a zoonotic disease seek veterinary treatment/management of their animal(s)?/Have you ever recommended a client who has an animal with or at risk of a zoonotic disease seek advice from a GP?’						
	Yes	No				
GPs^h^	118 (22.5%)	407 (77.5%)		1		
Veterinarians	491 (81.2%)	114 (18.8%)		18.05	12.90–25.25	<.001

^a^ Adjusted for rurality, gender, experience based on year of graduation, Australian university graduate and experience working in a developing country.

^b^ assumption of proportionality met.

^c^ Wald chi‐squared test.

^d^ 2 responses missing.

^e^ 1 response missing.

^f^ 5 responses missing.

^g^ 3 responses missing.

^h^ 3 responses missing.

Veterinarians had greater experience with zoonotic diseases than GPs; adjusted for other factors, the odds of a veterinarian having diagnosed a zoonotic disease were 9.36 times that of GPs (95% CI 6.31–13.87, *p* < .001; Table 3).

Adjusting for other factors, the odds of being more concerned about zoonoses in veterinarians were 2.3 times that of GPs (95% CI 1.76–3.11, *p* < .001). Practitioners from rural areas, those with more experience and those who had worked in developing countries were more concerned about zoonoses (Table S5). Veterinary practitioners working in small animal practice were found to have less concern than those in other areas of practice (Table S6).

Veterinarians were more confident in all areas than GPs; adjusted for other factors, the odds of a veterinarian being more confident about diagnosing, managing and giving advice about zoonoses were 14 times (95% CI 10.38–18.85, *p* < .001), 15 times (95% CI 11.14–20.29, *p* < .001) and 13 times (95% CI 9.71–17.36, *p* < .001) that of GPs, respectively. Rural practice and practitioner experience were also significant in the models (Table S5). Veterinary practitioner type played no difference in the level of practitioner confidence reported (Table S6).

For veterinarians, the odds of more frequently discussing the risk of zoonotic diseases or strategies to prevent them were around 5 times that of GPs, adjusting for other factors (95% CI 3.82–6.42, *p* < .001; Table 3). Rural practitioners and those who had worked in developing countries were more likely to discuss risk mitigation (Table S5), with small animal veterinarians being less likely to do so than other veterinarians (Table S6).

Finally, adjusting for other factors, veterinarians were around 18 times more likely to have recommended cross‐professional referral compared with GPs (95% CI 12.90–25.25, *p* < .001). Urban practitioners, those with greater experience and those who have worked in developed countries were more likely to refer (Table S5), as were small animal veterinarians (Table S6).

## 4 DISCUSSION

This distinctive, Australia‐wide study of GPs and veterinarians, highlighted significant differences in levels of experience, concern, confidence and practices with regard to zoonoses between practitioner groups. Veterinarians in clinical practice were found to more frequently diagnose zoonoses in their practice; have greater concern about zoonoses; be more confident in diagnosing, managing and giving advice about the prevention of zoonoses; more likely to give advice about managing risk of zoonoses; and more likely to initiate cross‐professional referral than GPs. Veterinarians’ greater clinical experience with zoonoses is reflected by a higher frequency of disease diagnoses in their practice. This is likely associated with their animal‐centred clinical focus. Their clinical exposure to zoonoses is likely to drive the greater concern, confidence and more regular zoonoses‐related practices reported in this study.

Heightened concern about zoonoses amongst veterinarians is likely to be propelled by a number of factors, primarily their greater knowledge of zoonoses and their epidemiology (Chaddock, 2012; Hennenfent et al., 2018; Hoff et al., 1999; John et al., 2008; Smout et al., 2017; Steele et al., 2018, 2019; Togami et al., 2018). Many veterinarians also have personal experience with zoonoses; nearly 45% of veterinarians in a previous Australian study reported contracting a zoonotic infection (Dowd et al., 2013). Veterinarians continuously make disease risk assessments as part of their daily professional practice, which potentially exposes them to serious zoonotic diseases (Dowd et al., 2013; Mendez et al., 2014). This was reflected in the list of diseases of concern where two fatal diseases (Hendra virus and ABLV) were listed in the top 8 diseases of concern for veterinarians, despite occurring infrequently compared with other endemic zoonoses. Additional concern may stem from the legal responsibility of veterinarians to manage risks to both animals and humans, including clients and staff, under local public health, biosecurity and work health and safety legislation and resulting fear of prosecution (Australian Veterinary Association, 2017; Mendez et al., 2012) . This was recently highlighted following successful civil cases against three veterinarians by horse owners in the case of Hendra virus (Buchanan, 2016). Concerns have also been raised by the professional association about the potential legal ramifications of Q fever infection of unvaccinated clients in veterinary practices (Australian Veterinary Association, 2018). All these factors are in contrast with GPs for whom patients with zoonoses are small component of their clinical practice (Australian College of Rural & Remote Medicine, 2007), most of whom present a low risk of infection to the GP and their staff.

GPs were found to be significantly less confident than veterinarians in diagnosing, managing and giving advice about the prevention of zoonoses. These findings were comparable with other survey‐based studies in the United States (Grant & Olsen, 1999; Hennenfent et al., 2018; Hill et al., 2012; von Matthiessen et al., 2003). This is thought by many to be a consequence of differing priorities and demands of medical and veterinary school curricula (Hodgson et al., 2019; John et al., 2008; Kahn et al., 2008; Natterson‐Horowitz, 2015; Rabinowitz & Conti, 2013; Togami et al., 2018), with medical graduates receiving less training in zoonoses, epidemiology and One Health (Chaddock, 2012; Hoff et al., 1999; John et al., 2008; Smout et al., 2017; Steele et al., 2018, 2019; Togami et al., 2018). The strategic position occupied by veterinarians and GPs in recognizing and reporting sentinel disease events (Morse et al., 2012; Rabinowitz & Conti, 2013) makes it imperative that educational interventions are put in place to improve the confidence, competence and capacity of GPs in this area. Our findings suggest that urban and less experienced GPs practitioners may be the highest priority for any such interventions.

The finding that GPs are less likely to discuss risk mitigation of zoonoses is likely to be a consequence of their different clinical experience of zoonoses (Grant & Olsen, 1999; Hill et al., 2012), lower levels of concern and confidence (Grant & Olsen, 1999; Kersting et al., 2009), and a less obvious clinical link to animal contact. Veterinary clinical training, incorporating assessment, discussion and mitigation of the risk of zoonoses (Rabinowitz & Conti, 2013) mean that veterinarians will more commonly address this with their clients. Rural practitioners, including rural GPs, were more likely to discuss risk reflecting more frequent presentations of clinically significant zoonoses, such as Q fever, leptospirosis, and brucellosis in rural areas or amongst rural workers.

Lower levels of experience, concern and confidence by GPs regarding zoonoses are probable causes of their less frequent engagement in cross‐professional referral practices. Historically, veterinarians have been more invested in the paradigm of One Health (Eussen et al., 2017; Gibbs & Gibbs, 2013; Marcotty et al., 2013; Speare et al., 2015), making endeavours to implement cross‐professional collaboration and co‐operation. This is also evident in the findings of this study. Both professions agree that cross‐professional collaboration is useful in managing zoonoses (Anholt et al., 2012; Hill et al., 2012; Speare et al., 2015); however, referral appears to happen infrequently using an ad hoc approach with little formal communication or follow‐up. Although low numbers, more than 10% of GPs used letters or phone calls when referring patients to veterinarians compared with less than 2% of veterinarians. This is probably a reflection of a more formalized system within medical practice of referring patients to other medical and allied health professionals with associated tracking in Medicare, Australia's publicly funded universal health insurance scheme (Department of Health, 2020).

Collaboration may also be impeded by time constraints, lack of understanding of health benefits and few established relationships between practitioner groups (Eussen et al., 2017; Grant & Olsen, 1999; von Matthiessen et al., 2003; Rabinowitz & Conti, 2013; Steele et al., 2019). Despite only 22% of GPs in this study recommending referral of a patient with, or at risk of, a zoonosis to a veterinarian, over two thirds of GPs said they would be willing to refer patients to a veterinarian who had extra training in zoonoses. This expressed willingness may reflect the usual model of GP referral to professionals with specific or specialized training, or perhaps interest in this pathway as a desirable option for future referrals. This finding is compatible with that of a previous Australian study where members of the public were willing to consult a veterinarian in the case of zoonoses on the recommendation of their physician (Speare et al., 2015) providing further support for the development of medico‐legal frameworks for cross‐professional referral. While the basic veterinary degree affords considerable training in zoonoses, further consideration could be given to post‐graduate qualifications. In Australia, Veterinary Public Health Master's degrees have tended to focus on risks, surveillance and response to animal disease outbreaks and food safety concerns (Toribio et al., 2009). Development of appropriate specialist qualifications through the Australian and New Zealand College of Veterinary Scientists in line with those of the American College of Veterinary Preventative Medicine may provide a suitably rigorous clinical qualification for veterinarians in the area of zoonoses and One Health. This may facilitate pathways for cross‐professional referral to veterinarians with advanced training in this area for management of animal‐based risk factors, especially in cases of zoonoses with significant human morbidity or mortality.

The emergence of SARS‐CoV‐2, alongside other historically recent outbreaks such as Ebola, Zika virus, H1N1 and SARS of zoonotic origin, highlights the need for all primary healthcare professionals to be confident clinically in the face of unusual disease events. However, the future risk of a disease outbreak with continuing zoonotic potential makes it imperative that a structured cross‐professional interface is established. Changes in government policy prioritizing further advancement of One Health practices in research, health education and governance, as well clinical practice, will enable effective multidisciplinary risk mitigation and response to emerging infectious diseases. This enhanced approach has potential to improve not just health outcomes, but also to reduce the economic impacts of large disease outbreaks. Gaps identified in this project will be further used to develop targeted joint educational interventions to build capacity and capability of GPs and veterinarians and explore pathways to facilitate cross‐professional relationships which will foster collaboration and referral with the ultimate aim of improving human and animal health outcomes in the area of zoonoses. Interventions for GPs, especially those from urban areas, specifically directed at improving knowledge of zoonotic risks are also indicated by the results of this study.

The main limitation of this study was the lower response rates of GPs, representing a smaller proportion of this practitioner group nationally, despite using a number of platforms to distribute online surveys. Additionally, GPs also had a higher rate of non‐completion. Both of these factors may contribute to non‐response bias which is a frequently encountered issue when conducting surveys amongst medical practitioners (Brodaty et al., 2013; Kellerman & Herold, 2001; Scott et al., 2011) and appears unrelated to the method of data collection. However, a representative sample was achieved, with sufficient numbers recruited from both practitioner groups. Open‐ended questions asked in the survey did not explore reasons for levels of practitioner confidence and differences in referral practices. Additionally, drivers of practitioner concern are complex and dependent on a number of interplaying factors. Assessment of these was beyond the scope of this study and is areas that may benefit from a deeper exploration of ideas using interviews or focus groups which will be addressed in future research.

## 5 CONCLUSION

Both GPs and veterinarians play a vital role in primary health care and disease surveillance in humans and animals, respectively. Australian veterinarians showed greater experience and concern and were more confidence with managing zoonoses than their GP counterparts. Also, they were more likely to engage in discussion about the risk of zoonoses and to refer their clients to GPs on the suspicion of a zoonosis or to mitigate the risk of zoonoses. The findings of this study indicate a need for change in both medical and veterinary education as well as continuing professional education, especially for GPs, in order to better equip them in the area of zoonoses. Exploration of pathways to encourage and facilitate cross‐professional referral and collaboration will further improve clinical outcomes for both human and animals.

## CONFLICT OF INTEREST

The authors declare they have no conflict of interest in relation to this paper.

## ETHICAL APPROVAL

Approval for the project was granted by the University of Sydney human ethics committee (project number 2018/980).

## Supporting information

Tables S1–S6

## APPENDIX 1

### GP Survey

What is the postcode(s) of your primary workplace?

What is your gender?

- Male
- Female
- Transgender
- Not listed
- Prefer not to say

At which university did you complete your medical degree?

In what area of medical practice do you work?

- General practice
- Other (Please specify)

What year did you graduate from your medical degree?

Do you have any qualifications in addition to your medical degree? Y/N

Please specify

- Master's degree
  - FRACGP or overseas equivalent
- PhD
- Other (Please specify)

Have you ever worked in:

- A rural or remote area Y/N
- A developing country Y/N

How concerned are you about zoonotic diseases in the practice you currently work in?

- Not concerned
- Somewhat concerned
- Moderately concerned
- Very concerned
- Extremely concerned

Which zoonotic disease concerns you the most in the practice you currently work in?

Have you noticed any changes in the patterns of zoonotic disease occurrence in your region that No could be associated with environmental changes? Y/N

Please comment if desired

In what clinical situations (if any) would you ask patients about contact with animals?

Would you ask about animal contact in the context of a patient with diarrhoea and/or vomiting?

Y/N

What disease(s) would you be concerned about?

How often do you discuss potential risk of zoonotic diseases or strategies to prevent these with your patients?

- Never
- Occasionally
- Sometimes
- Frequently
- Always

What factors would prompt you to do this?

Have you ever diagnosed a zoonotic disease in a patient? Y/N

Please list all zoonotic diseases you have diagnosed in your current job

Have you ever recommended a patient with, or at risk of, a zoonotic disease seek veterinary treatment/management of their animal(s)? Y/N

What were the circumstances?

How did you do the referral in this case?

- Verbal recommendation to patient
- Verbal recommendation and asked patient to get vet to ring you
- Business card given to patient to give their veterinarian
- Phone call to veterinarian directly
- Wrote referral letter
- Wrote referral letter with specific recommendations
- Other (Please specify)

Have you ever had a patient referred to you by a veterinarian due to:

- exposure to a diagnosed or suspected zoonotic disease Y/N
- concern about risk factors for a zoonotic disease Y/N
- an animal‐related injury Y/N

Please specify

Would you consider referring your patients to a veterinarian with extra training in zoonotic diseases for advice about reducing risk of infection with zoonoses or potential zoonoses? Y/N

Overall, how confident do you feel in your ability to:

- Diagnose common zoonotic diseases
- Manage common zoonotic diseases
- Give advice about prevention of common zoonotic diseases
- Not confident
- Somewhat confident
- Moderately confident
- Confident
- Very confident

### Veterinarian Survey

What is the postcode(s) of your primary workplace?

What is your gender?

- Male
- Female
- Transgender
- Not listed
- Prefer not to say

At which university did you complete your veterinary degree?

In what area of veterinary practice do you work?

- Small animal practice
- Mixed practice
- Equine practice
- Exotics practice
- Avian practice
- Other (Please specify)

What year did you graduate from your veterinary degree?

Do you have any qualifications in addition to your veterinary degree?

- Master's degree
- Memberships or overseas equivalent
- Fellowships or overseas equivalent
- PhD
- Other (Please specify)

(please tick all that apply)

Have you ever worked in:

a) A rural or remote area Y/N

b) A developing country Y/N

How concerned are you about zoonotic diseases in the practice you currently work in?

- Not concerned
- Somewhat concerned
- Moderately concerned
- Very concerned
- Extremely concerned

Which zoonotic disease concerns you the most in the practice you currently work in?

Have you noticed any changes in the patterns of zoonotic disease occurrence in your region that No could be associated with environmental changes? Y/N

Please comment if desired

In what clinical situations (if any) would you ask about the home environment and household contacts of an animal?

How often do you discuss potential risk of zoonotic diseases or strategies to prevent these with your clients?

- Never
- Occasionally
- Sometimes
- Frequently
- Always

What factors would prompt you to do this?

Have you ever diagnosed a zoonotic disease in an animal? Y/N

Please list all zoonotic diseases you have diagnosed in your current job

Have you ever recommended a client who has an animal with or at risk of a zoonotic disease seek advice from a GP? Y/N

What were the circumstances? __________________________________________

How did you do the referral in this case?

- Verbal recommendation to client
- Verbal recommendation and asked client to get GP to ring you
- Business card given to patient to give their GP
- Phone call to GP directly
- Wrote referral letter
- Wrote referral letter with specific recommendations
- Other (Please specify)

Have you ever had a client referred to you by a doctor for assessment or management of an animal due to:

- exposure to a diagnosed or suspected zoonotic disease
- concern about risk factors for a zoonotic disease

Please specify

Overall, how confident do you feel in your ability to:

- Diagnose common zoonotic diseases
- Manage common zoonotic diseases
- Give advice about prevention of common zoonotic diseases
- Not confident
- Somewhat confident
- Moderately confident
- Confident
- Very confident

## References

[zph12799-bib-0001] AHPRA (2019). Medical Board of Australia Registrant data, Reporting period: 01 July 2019 to 30 September 2019. Statistics, Registration Data. Retrieved from https://www.medicalboard.gov.au/News/Statistics.aspx

[zph12799-bib-0002] Anholt, R. M. , Stephen, C. , & Copes, R. (2012). Strategies for collaboration in the interdisciplinary field of emerging zoonotic diseases. Zoonoses & Public Health, 59(4), 229–240. 10.1111/j.1863-2378.2011.01449.x 22273426

[zph12799-bib-0003] Animal Medicines Australia (2019). Pets in Australia: A national survey of pets and people. Retrieved from https://animalmedicinesaustralia.org.au/report/pets‐in‐australia‐a‐national‐survey‐of‐pets‐and‐people/

[zph12799-bib-0004] Annand, E. J. , & Reid, P. A. (2014). Clinical review of two fatal equine cases of infection with the insectivorous bat strain of Australian bat lyssavirus. Australian Veterinary Journal, 92(9), 324–332. 10.1111/avj.12227 25156050

[zph12799-bib-0005] Australian Bureau of Statistics (2018). Postcode 2017 to Remoteness Area 2016. Retrieved from https://www.abs.gov.au/AUSSTATS/abs@.nsf/DetailsPage/1270.0.55.005July%202016?OpenDocument

[zph12799-bib-0006] Australian Bureau of Statistics (2019, 28 May 2020). 7121.0 ‐ Agricultural Commodities, Australia, 2018–19. Retrieved from https://www.abs.gov.au/Ausstats/abs@.nsf/7d12b0f6763c78caca257061001cc588/6f2e0b4aa8c0b248ca25815500117f18!OpenDocument#:~:text=As%20at%2030%20June%202019,other%20cattle%20(down%209%25)

[zph12799-bib-0007] Australian College of Rural and Remote Medicine (2007). Position Statement: Defining the specialty of General Practice. Retrieved from https://www.acrrm.org.au/docs/default‐source/documents/about‐the‐college/position‐statement‐defining‐the‐specialty‐of‐general‐practice.pdf?sfvrsn=e99a90eb_4

[zph12799-bib-0008] Australian Medical Association (2017). Australian National Centre for Disease Control (CDC) ‐ 2017. Retrieved from https://ama.com.au/position‐statement/australian‐national‐centre‐disease‐control‐cdc‐2017

[zph12799-bib-0009] Australian Veterinary Association (2017) Guidelines for Veterinary Personal Biosecurity 2017 (3rd edn). Australian Veterinary Association.

[zph12799-bib-0010] Australian Veterinary Association (2018, 7 December 2018). Q Fever protection in veterinary practice. Professional practices for veterinarians. Retrieved from https://www.ava.com.au/policy‐advocacy/policies/professional‐practices‐for‐veterinarians/q‐fever‐protection‐in‐veterinary‐practice/

[zph12799-bib-0011] Australian Veterinary Boards Council (2019). Search for registered veterinarians in Australia. Retrieved from https://avbc.asn.au/search‐for‐registered‐vet/

[zph12799-bib-0012] Brodaty, H. , Gibson, L. H. R. , Waine, M. L. , Shell, A. M. , Lilian, R. , & Pond, C. D. (2013). Research in general practice: A survey of incentives and disincentives for research participation. Mental Health in Family Medicine, 10(3), 163–173. 24427184 PMC3822664

[zph12799-bib-0013] Buchanan, K. (2016, 16 September 2016). Queensland vet found guilty of breaching workplace laws while treating a horse with Hendra virus. *ABC News*. Retrieved from https://www.abc.net.au/news/rural/2016‐09‐30/vet‐found‐guilty‐over‐hendra‐case/7892316#:~:text=An%20Olympic%20equine%20vet%20has,Section%2028%20of%20the%20act

[zph12799-bib-0014] Chaddock, M. (2012). Academic Veterinary Medicine and One Health Education: It Is more than Clinical Applications. Journal of Veterinary Medical Education, 39(3), 241–246. 10.3138/jvme.0612-062 22940444

[zph12799-bib-0015] Chomel, B. , & Sun, B. (2011). Zoonoses in the bedroom. Emerging Infectious Diseases, 17(2), 167–172. 10.3201/eid1702101070 21291584 PMC3298380

[zph12799-bib-0016] Clutterbuck, H. C. , Eastwood, K. , Massey, P. D. , Hope, K. , & Mor, S. M. (2018). Surveillance system enhancements for Q fever in NSW, 2005–2015. Communicable Disease Intelligence, 2018, 42. 10.33321/cdi.2018.42.10 30626297

[zph12799-bib-0017] Department of Health (2019a, 7 August 2019). The Australian health system. Retrieved from https://www.health.gov.au/about‐us/the‐australian‐health‐system

[zph12799-bib-0018] Department of Health (2019b). National Notifiable Diseases Surveillance System. Retrieved from http://www9.health.gov.au/cda/source/rpt_2.cfm

[zph12799-bib-0019] Department of Health (2020). Medicare. Retrieved from https://www.health.gov.au/health‐topics/medicare

[zph12799-bib-0020] Dowd, K. , Taylor, M. , Toribio, J.‐A.‐L.‐M.‐L. , Hooker, C. , & Dhand, N. K. (2013). Zoonotic disease risk perceptions and infection control practices of Australian veterinarians: Call for change in work culture. Preventive Veterinary Medicine, 111(1–2), 17–24. 10.1016/j.prevetmed.2013.04.002 23664739 PMC7127186

[zph12799-bib-0021] Eastwood, K. , Massey, P. D. , Hutchinson, P. , van den Berg, D. , Bosward, K. , & Graves, S. R. (2018). Q fever: A rural disease with potential urban consequences. Australian Journal of General Practice, 47(3), 112–115. 10.31128/AFP-08-17-4299 29621839

[zph12799-bib-0022] Eussen, B. G. M. , Schaveling, J. , Dragt, M. J. , & Blomme, R. J. (2017). Stimulating collaboration between human and veterinary health care professionals. BMC Veterinary Research, 13(1), 174. 10.1186/s12917-017-1072-x 28610617 PMC5470326

[zph12799-bib-0023] Gibbs, S. E. , & Gibbs, E. P. (2013). The historical, present, and future role of veterinarians in One Health. Current Topics in Microbiology & Immunology, 365, 31–47. 10.1007/82_2012_259 22911439 PMC7121980

[zph12799-bib-0024] Grant, S. , & Olsen, C. W. (1999). Preventing zoonotic diseases in immunocompromised persons: The role of physicians and veterinarians. Emerging Infectious Diseases, 5(1), 159–163. 10.3201/eid0501.990121 10081686 PMC2627689

[zph12799-bib-0025] Heiberger, R. M. , & Heiberger, M. R. M. (2017). HH: Statistical Analysis and Data Display. In *Springer Texts in Statistics*. Retrieved from https://CRAN.R‐project.org/package=HH

[zph12799-bib-0026] Heiberger, R. , & Robbins, N. (2014). Design of Diverging Stacked Bar Charts for Likert Scales and Other Applications. Journal of Statistical Software, 57(5), 1–32 10.18637/jss.v057.i05.25400517

[zph12799-bib-0027] Hennenfent, A. K. , Iyengar, P. , & Davies‐Cole, J. (2018). Assessing rabies knowledge gaps in human and animal healthcare professionals practicing in Washington, DC‐A one health approach. Zoonoses & Public Health, 65(8), 947–956. 10.1111/zph.12514 30099849

[zph12799-bib-0028] Hill, W. A. , Petty, G. C. , Erwin, P. C. , & Souza, M. J. (2012). A survey of Tennessee veterinarian and physician attitudes, knowledge, and practices regarding zoonoses prevention among animal owners with HIV infection or AIDS. Journal of the American Veterinary Medical Association, 240(12), 1432–1440. 10.2460/javma.240.12.1432 22657926

[zph12799-bib-0029] Hodgson, K. , Darling, M. , Freeman, D. , & Monavvari, A. (2019). Engaging family physicians in one health. Journal of the American Veterinary Medical Association, 254(11), 1267–1269. 10.2460/javma.254.11.1267 31067186

[zph12799-bib-0030] Hoff, G. L. , Brawley, J. , & Johnson, K. (1999). Companion animal issues and the physician. Southern Medical Journal, 92(7), 651–659. 10.1097/00007611-199907000-00002 10414472

[zph12799-bib-0031] John, K. , Kazwala, R. , & Mfinanga, G. S. (2008). Knowledge of causes, clinical features and diagnosis of common zoonoses among medical practitioners in Tanzania. BMC Infectious Diseases, 8(1), 162. 10.1186/1471-2334-8-162 19046464 PMC2611996

[zph12799-bib-0032] Jones, K. E. , Patel, N. G. , Levy, M. A. , Storeygard, A. , Balk, D. , Gittleman, J. L. , & Daszak, P. (2008). Global trends in emerging infectious diseases. Nature, 451(7181), 990–993. 10.1038/nature06536 18288193 PMC5960580

[zph12799-bib-0033] Kahn, L. H. , Kaplan, B. , Monath, T. P. , & Steele, J. H. (2008). Teaching “one medicine, one health”. American Journal of Medicine, 121, 10.1016/j.amjmed.2007.09.023 PMC7119384 18328295

[zph12799-bib-0034] Kahn, L. H. , Kaplan, B. , & Steele, J. H. (2007). Confronting zoonoses through closer collaboration between medicine and veterinary medicine (as 'one medicine'). Veterinaria Italiana, 43(1), 5–19. 20411497

[zph12799-bib-0035] Kellerman, S. E. , & Herold, J. (2001). Physician response to surveys. A review of the literature. American Journal of Preventive Medicine, 20(1), 61–67. 10.1016/S0749-3797(00)00258-0 11137777

[zph12799-bib-0036] Kersting, A. L. , Medeiros, L. C. , & LeJeune, J. T. (2009). Zoonoses and the physicians' role in educating farming patients. Journal of Agromedicine, 14(3), 306–311. 10.1080/10599240903058160 19657880

[zph12799-bib-0037] Mahalingam, S. , Herrero, L. J. , Playford, E. G. , Spann, K. , Herring, B. , Rolph, M. S. , Middleton, D. , McCall, B. , Field, H. , & Wang, L.‐F. (2012). Hendra virus: An emerging paramyxovirus in Australia. The Lancet Infectious Diseases, 12(10), 799–807. 10.1016/S1473-3099(12)70158-5 22921953

[zph12799-bib-0038] Marcotty, T. , Thys, E. , Conrad, P. , Godfroid, J. , Craig, P. , Zinsstag, J. , Meheus, F. , Boukary, A. R. , Badé, M. A. , Sahibi, H. , Filali, H. , Hendrickx, S. , Pissang, C. , Van Herp, M. , van der Roost, D. , Thys, S. , Hendrickx, D. , Claes, M. , Demeulenaere, T. , … Boelaert, M. (2013). Intersectoral collaboration between the medical and veterinary professions in low‐resource societies: The role of research and training institutions. Comparative Immunology, Microbiology and Infectious Diseases, 36(3), 233–239. 10.1016/j.cimid.2012.10.009 23260374

[zph12799-bib-0039] Mendez, D. , Buttner, P. , & Speare, R. (2014). Hendra virus in Queensland, Australia, during the winter of 2011: Veterinarians on the path to better management strategies. Preventive Veterinary Medicine, 117(1), 40–51. 10.1016/j.prevetmed.2014.08.002 25175674 PMC7132398

[zph12799-bib-0040] Mendez, D. , Judd, J. , & Speare, R. (2012). Unexpected result of Hendra virus outbreaks for veterinarians, Queensland. Australia. Emerging Infectious Diseases, 18(1), 83–85. 10.3201/eid1801.111006 22261152 PMC3310112

[zph12799-bib-0041] Morse, S. S. , Mazet, J. A. K. , Woolhouse, M. , Parrish, C. R. , Carroll, D. , Karesh, W. B. , Zambrana‐Torrelio, C. , Lipkin, W. I. , & Daszak, P. (2012). Prediction and prevention of the next pandemic zoonosis. Lancet, 380(9857), 1956–1965. 10.1016/s0140-6736(12)61684-5 23200504 PMC3712877

[zph12799-bib-0042] Natterson‐Horowitz, B. (2015). A Physician's View of One Health: Challenges and Opportunities. Veterinary Sciences, 2(1), 23–25. 10.3390/vetsci2010023 29061927 PMC5644612

[zph12799-bib-0043] Rabinowitz, P. , & Conti, L. (2010). Human‐Animal Medicine: Clinical Approaches to Zoonoses, Toxicants and Other Shared Health Risks. Saunders Elsevier.

[zph12799-bib-0044] Rabinowitz, P. , & Conti, L. (2013). One Health and Emerging Infectious Diseases: Clinical Perspectives. In J. S. Mackenzie , M. Jeggo , P. Daszak , & J. A. Richt (Eds.), One Health: The human‐animal‐environment interfaces in emerging infectious diseases: The concept and examples of a One Health Approach (pp. 17–29). Springer. 10.1007/82_2012_263 PMC7122511 22976348

[zph12799-bib-0045] Scott, A. , Jeon, S.‐H. , Joyce, C. M. , Humphreys, J. S. , Kalb, G. , Witt, J. , & Leahy, A. (2011). A randomised trial and economic evaluation of the effect of response mode on response rate, response bias, and item non‐response in a survey of doctors. BMC Medical Research Methodology, 11(1), 126. 10.1186/1471-2288-11-126 21888678 PMC3231767

[zph12799-bib-0046] Shomaker, T. S. , Green, E. M. , & Yandow, S. M. (2013). Perspective: One health: A compelling convergence. Academic Medicine, 88, 10.1097/ACM.0b013e31827651b1 23165268

[zph12799-bib-0047] Smout, F. , Schrieber, L. , Speare, R. , & Skerratt, L. F. (2017). More bark than bite: Comparative studies are needed to determine the importance of canine zoonoses in Aboriginal communities. A critical review of published research. Zoonoses & Public Health, 64(7), 495–504. 10.1111/zph.12354 28342271 PMC7159129

[zph12799-bib-0048] Speare, R. , Mendez, D. , Judd, J. , Reid, S. , Tzipori, S. , & Massey, P. D. (2015). Willingness to Consult a Veterinarian on Physician’s Advice for Zoonotic Diseases: A Formal Role for Veterinarians in Medicine? PLoS One, 10(8), e0131406. 10.1371/journal.pone.0131406 26237399 PMC4523201

[zph12799-bib-0049] Steele, S. G. , Booy, R. , & Mor, S. M. (2018). Establishing research priorities to improve the One Health efficacy of Australian general practitioners and veterinarians with regard to zoonoses: A modified Delphi survey. One Health, 6, 7–15. 10.1016/j.onehlt.2018.08.001 30197925 PMC6127845

[zph12799-bib-0050] Steele, S. G. , Toribio, J.‐A. , Booy, R. , & Mor, S. M. (2019). What makes an effective One Health clinical practitioner? Opinions of Australian One Health experts. One Health, 8, 100108. 10.1016/j.onehlt.2019.100108 31720358 PMC6838466

[zph12799-bib-0051] Togami, E. , Gardy, J. L. , Hansen, G. R. , Poste, G. H. , Rizzo, D. M. , Wilson, M. E. , & Mazet, J. A. K. (2018). Core Competencies in One Health Education: What Are We Missing? NAM Perspectives, 8(6), 1–12. 10.31478/201806a.

[zph12799-bib-0052] Toribio, J. A. , Forsyth, H. , Laxton, R. , & Whittington, R. J. (2009). An innovative approach to post‐graduate education in veterinary public health. Journal of Veterinary Medical Education, 36(1), 114–121. 10.3138/jvme.36.1.114 19435998

[zph12799-bib-0053] von Matthiessen, P. W. , Sansone, R. A. , Meier, B. P. , Gaither, G. A. , & Shrader, J. (2003). Zoonotic diseases and at‐risk patients: A survey of veterinarians and physicians. Aids, 17(9), 1404–1406. 10.1097/00002030-200306130-00021 12799568

[zph12799-bib-0054] Wang, L. F. (2011). Discovering novel zoonotic viruses. New South Wales Public Health Bulletin, 22(5–6), 113–117. 10.1071/NB10078 21781618

