# Supplementary material for: Towards One Health clinical management of zoonoses: A parallel survey of Australian general medical practitioners and veterinarians
Source: Zoonoses Public Health. 2020 Dec 31;68(2):88–102. doi: 10.1111/zph.12799 (PMC7986233; doi:10.1111/zph.12799)
Supplement: Supplementary file 1 — Tables S1–S6 [file ZPH-68-88-s001.docx]

**Table S1: Modes of recruitment for online surveys of Australian general medical practitioners (GPs) and veterinarians.**

| **Organisation** | **Distribution** |
| --- | --- |
| **General practitioners were notified by:** | |
| Healthed | Email list, webinar |
| Primary Health Networks – all were contacted by phone, approximately 1/3 agreed to distribute the survey link. | Online newsletters, website item |
| Australian Medical Association | GP online newsletter. |
| GPs Down Under Facebook group | Link was shared on an Australian GP Facebook group through word of mouth. |
| **Veterinarians were notified by:** | |
| Australian Veterinary Association | Personal invitation by researcher at national conference using business cards with Q-R code link, tablets available for participant use.  Distribution in AVA national newsletter and some special interest group newsletters. |
| Australian and New Zealand College of Veterinary Scientists | Personal invitation by researcher at Science Week conference using business cards with Q-R code link, tablets available for participant use. |
| Australian Veterinary Network Facebook Group | Link was shared by researcher in post(s) |
| Australian Veterinarians in Public Health FB group | Link was shared by researcher in post(s) |
| Australian/NZ Wildlife, Avian and Exotics Network FB group | Link was shared by researcher in post(s) |
| University of Sydney Centre for Continuing Veterinary Education | Newsletter |
| Veterinary Practitioner boards in NSW, ACT, Vic, Tas, NT and WA. | Newsletter and/or email. |

**Table S2.** **Responses by Australian general medical practitioners (GPs; n=528) and veterinarians (n=605) regarding clinician experience, concern, confidence and practices related to zoonoses.**

|  |  | GP | | Veterinarian | | P value |
| --- | --- | --- | --- | --- | --- | --- |
|  |  | N | % | N | % |  |
| Have you ever diagnosed a zoonotic disease in a patient? ^e^ | Yes | 316 | **60.1** | 564 | **93.2** | <0.001 |
| How concerned are you about zoonotic diseases in the practice you currently work in? ^a^ | Not concerned | 178 | **33.8** | 87 | **14.4** | <0.001 |
|  | Somewhat concerned | 224 | **42.5** | 268 | **44.3** |  |
|  | Moderately concerned | 94 | **17.8** | 185 | **30.6** |  |
|  | Very concerned | 27 | **5.1** | 53 | **8.8** |  |
|  | Extremely concerned | 4 | **0.8** | 12 | **2.0** |  |
| Overall, how confident do you feel in your ability to: |  |  |  |  |  |  |
| Diagnose common zoonotic diseases ^b,c^ | Not confident | 178 | **34.0** | 5 | **0.8** | <0.001 |
|  | Somewhat confident | 208 | **39.8** | 122 | **20.3** |  |
|  | Moderately confident | 105 | **20.1** | 245 | **40.7** |  |
|  | Confident | 26 | **5.0** | 195 | **32.4** |  |
|  | Very confident | 6 | **1.1** | 35 | **5.8** |  |
| Manage common zoonotic diseases ^b,c^ | not confident | 168 | **32.1** | 15 | **2.5** | <0.001 |
|  | somewhat confident | 207 | **39.6** | 99 | **16.4** |  |
|  | moderately confident | 113 | **21.6** | 234 | **38.9** |  |
|  | confident | 29 | **5.5** | 214 | **35.6** |  |
|  | very confident | 6 | **1.1** | 40 | **6.6** |  |
| Give advice about prevention of common zoonotic diseases ^b,c^ | not confident | 156 | **29.8** | 5 | **0.8** | <0.001 |
|  | somewhat confident | 199 | **38.0** | 94 | **15.6** |  |
|  | moderately confident | 119 | **22.8** | 217 | **36.0** |  |
|  | confident | 44 | **8.4** | 233 | **38.7** |  |
|  | very confident | 5 | **1.0** | 53 | **8.8** |  |
| How often do you discuss potential risk of zoonotic diseases or strategies to prevent these with your patients/clients? | never | 80 | **15.2** | 7 | **1.2** | <0.001 |
|  | occasionally | 256 | **48.5** | 172 | **28.4** |  |
|  | sometimes | 141 | **26.7** | 201 | **33.2** |  |
|  | frequently | 49 | **9.3** | 191 | **31.6** |  |
|  | always | 2 | **0.4** | 34 | **5.6** |  |
| Have you ever recommended a patient with or at risk of a zoonotic disease seek veterinary treatment/management of their animal(s)? / Have you ever recommended a client who has an animal with or at risk of a zoonotic disease seek advice from a GP? ^d^ | Yes | 118 | **22.5** | 491 | **81.2** | <0.001 |
|  | No | 407 | **77.5** | 114 | **18.8** |  |
| How did you do the referral in this case? | Verbal recommendation to client | 93 | **78.8** | 448 | **91.2** |  |
|  | Verbal recommendation and asked client to get veterinarian/GP to ring you | 5 | **4.2** | 15 | **3.1** |  |
|  | Business card given to client to give to their veterinarian/GP | 2 | **1.7** | 7 | **1.4** |  |
|  | Phone call to veterinarian/GP directly | 4 | **3.4** | 7 | **1.4** |  |
|  | Wrote referral letter | 10 | **8.5** | 2 | **0.4** |  |
|  | Wrote referral letter with specific recommendations | 1 | **0.85** | 1 | **0.2** |  |
|  | Other (please specify) | 2 | **1.7** | 15 | **3.1** |  |
| Would you consider referring your patients to a veterinarian with extra training in zoonotic diseases for advice about reducing risk of infection with zoonoses or potential zoonoses? (GP) | Yes | 354 | **67.7** |  |  |  |
|  | No | 169 | **32.3** |  |  |  |
| Have you ever had a patient referred to you by a veterinarian/GP due to: |  |  |  |  |  |  |
| Exposure to a diagnosed or suspected zoonotic disease ^a^ | Yes | 32 | **6.1** | 205 | **33.9** | <0.001 |
|  | No | 492 | **93.9** | 400 | **66.1** |  |
| Concern about risk factors for a zoonotic disease ^e,f^ | Yes | 25 | **4.8** | 140 | **23.3** | <0.001 |
|  | No | 498 | **95.2** | 462 | **76.4** |  |
| An animal related injury ^g^ | Yes | 91 | **17.6** |  |  |  |
|  | No | 431 | **82.4** |  |  |  |

a: 1 GP response missing

b :5 GP responses missing

c: 3 vet responses missing

d: 3 GP responses missing

e: 2 GP responses missing

f: 3 vet responses missing

g: 6 GP responses missing

**Table S3: Top 20 most commonly diagnosed zoonoses, as reported by Australian general medical practitioners (GPs; n=316) and veterinarians (n=564) who had ever diagnosed a zoonosis in a patient.**

| Disease/Agent | N | % |
| --- | --- | --- |
| Top 20 zoonoses diagnosed: GPs | | |
| Bacterial Gastroenteritis^†^ | 148 | **46.8** |
| Q fever | 114 | **36.1** |
| Ringworm (Dermatophytosis) | 70 | **22.2** |
| Giardia spp | 54 | **17.1** |
| Leptospirosis (*Leptospira spp*) | 51 | **16.1** |
| Ross River Fever | 46 | **14.6** |
| Toxoplasmosis (*Toxoplasma gondii*) | 37 | **11.7** |
| *Chlamydia Psittaci* | 34 | **10.8** |
| Cat scratch fever (*Bartonella henselae*) | 29 | **9.2** |
| Tick typhus (*Rickettsia spp)* | 29 | **9.2** |
| Brucellosis (*B. suis)* | 28 | **8.9** |
| Animal Bites | 28 | **8.9** |
| Dengue fever | 28 | **8.9** |
| Malaria | 26 | **8.9** |
| Influenza ^‡^ | 22 | **8.2** |
| Orf | 21 | **7.0** |
| Barmah Forest Virus | 16 | **6.6** |
| Common zoonotic endoparasites ^§^ | 28 | **5.1** |
| Tick and flea bites | 14 | **4.4** |
| *Echinococcus granulosus* | 12 | **3.8** |
|  |  |  |
| Top 20 zoonoses diagnosed: veterinarians | | |
| Bacterial Gastroenteritis ^†^ | 488 | **86.5** |
| Ringworm (Dermatophytosis) | 439 | **77.8** |
| Toxoplasmosis (*Toxoplasma gondii*) | 149 | **26.4** |
| Common zoonotic endoparasites ^§^ | 126 | **22.3** |
| *Sarcoptes scabeii* | 113 | **20.0** |
| Giardia spp | 106 | **18.8** |
| *Chlamydia Psittaci* | 102 | **18.1** |
| Leptospirosis (*Leptospira spp*) | 92 | **16.3** |
| MRSA/MRSP | 55 | **9.8** |
| cryptosporidium | 41 | **7.3** |
| Brucellosis | 36 | **6.4** |
| Cryptococcus spp | 24 | **4.3** |
| *Mycobacterium avium* | 23 | **4.1** |
| Coccidiosis | 17 | **3.0** |
| Orf | 14 | **2.5** |
| *Echinococcus granulosus* | 14 | **2.5** |
| Q fever (*Coxiella burnetti*) | 12 | **2.1** |
| ABLV/bat viruses | 11 | **2.0** |
| *Bordatella bronchiseptica* | 11 | **2.0** |
| Influenza ^‡^ | 10 | **1.8** |

†includes *Campylobacter* spp, *Salmonella* spp and *E. coli*

‡ includes H5N1 and H1N1.

§ includes parasites causing visceral larva migrans (*Toxocara* spp) and cutaneous larva migrans (*Ancyclostoma* spp) and flea tapeworm (*Dipylidium caninum*)

**Table S4: Top 20 zoonoses of concern, as reported by Australian general medical practitioners (GPs; n=349) and veterinarians (n=518) who expressed any level of concern about zoonoses.**

| Disease/Agent | N | % |
| --- | --- | --- |
| Top 20 zoonoses of concern: GPs | | |
| Q fever (*Coxiella burnetti*) | 174 | **49.9** |
| Bacterial Gastroenteritis ^†^ | 85 | **24.4** |
| Rabies | 52 | **14.9** |
| Toxoplasmosis (*Toxoplasma gondii*) | 49 | **14.0** |
| Leptospirosis (*Leptospira spp*) | 45 | **12.9** |
| Ross River Fever | 44 | **12.6** |
| Giardia spp | 37 | **10.6** |
| Travel related viral zoonoses^‡^ | 37 | **10.6** |
| Influenza ^§^ | 35 | **10.0** |
| Animal bites | 35 | **10.0** |
| ABLV/bat viruses | 34 | **9.7** |
| Tick borne diseases | 33 | **9.5** |
| Common zoonotic endoparasites ^¶^ | 29 | **8.3** |
| Ringworm (Dermatophytosis) | 28 | **8.0** |
| *Chlamydia Psittaci* | 28 | **8.0** |
| Brucellosis | 24 | **6.9** |
| Dengue fever | 23 | **6.6** |
| Malaria | 23 | **6.6** |
| Listeriosis | 21 | **6.0** |
| Mosquito borne diseases | 18 | **5.2** |
|  |  |  |
| Top 20 zoonoses of concern: veterinarians | | |
| Ringworm (Dermatophytosis) | 232 | **44.8** |
| Bacterial Gastroenteritis ^†^ | 220 | **42.5** |
| Q fever (*Coxiella burnetti*) | 144 | **27.8** |
| *Chlamydia Psittaci* | 110 | **21.2** |
| Toxoplasmosis (*Toxoplasma gondii*) | 96 | **18.5** |
| Leptospirosis (*Leptospira spp*) | 91 | **17.6** |
| Hendra virus | 81 | **15.6** |
| Australian Bat Lyssavirus | 73 | **14.1** |
| Common zoonotic endoparasites ¶ | 68 | **13.1** |
| Brucellosis | 54 | **10.4** |
| MRSA/MRSP | 53 | **10.2** |
| Giardia spp | 45 | **8.7** |
| Bartonellosis/cat scratch fever | 44 | **8.5** |
| Animal bites | 33 | **6.4** |
| Multi-drug resistant organisms | 33 | **6.4** |
| *Sarcoptes scabeii* | 31 | **6.0** |
| *Echinococcus granulosus* | 19 | **3.7** |
| Cryptosporidium | 16 | **3.1** |
| Anthrax | 10 | **1.9** |
| Rabies | 9 | **2.0** |

† includes *Campylobacter* spp, *Salmonella* spp and *E. coli*

‡ includes Ebola virus and Zika virus

§ includes H5N1 and H1N1.

¶ includes parasites causing visceral larva migrans (*Toxocara* spp) and cutaneous larva migrans (*Ancyclostoma* spp) and flea tapeworm (*Dipylidium caninum*)

**Table S5: Results of multivariable analysis (full models) comparing responses of Australian general medical practitioners (GPs; n=528) and veterinarians (n=605) who participated in the online surveys.**

| Explanatory Variable | *b* | SE | p |
| --- | --- | --- | --- |
| Experience with zoonotic diseases  ‘Have you ever diagnosed a zoonotic disease in a patient?’ | | | |
| Veterinarian v GP | 2.23 | 0.20 | <0.001 |
| Rural v urban | 0.17 | 0.17 | 0.436 |
| Male v female | -0.10 | 0.18 | 0.488 |
| Year of graduation |  |  | 0.078 |
| 2010-2019 | 0 |  |  |
| 2000-2009 | 0.56 | 0.26 |  |
| 1990-1999 | 0.58 | 0.27 |  |
| <1989 | 0.27 | 0.24 |  |
| Australian v overseas graduate | 0.05 | 0.22 | 0.987 |
| Developing country v other | 0.13 | 0.20 | 0.519 |
|  | | | |
| Model 1: Concern  ‘How concerned are you about zoonotic diseases in the practice you currently work in?’ | | | |
| Veterinarian v GP | 0.85 | 0.14 | <0.001 |
| Rural v urban | 0.76 | 0.13 | <0.001 |
| Male v female | 0.35 | 0.15 | 0.020 |
| Year of graduation |  |  | 0.542 |
| 2010-2019 | 0 |  |  |
| 2000-2009 | -0.14 | 0.19 |  |
| 1990-1999 | 0.25 | 0.20 |  |
| <1989 | 0.09 | 0.19 |  |
| Australian v overseas graduate | 0.20 | 0.19 | 0.29 |
| Developing country v other | 0.40 | 0.16 | 0.012 |
|  |  |  |  |
| Model 2: Confidence  ‘Overall, how confident do you feel in your ability to:’ | | | |
| 2a: Diagnosis  ‘Diagnose common zoonotic diseases’ | | | |
| Veterinarian v GP | 2.64 | 0.15 | <0.001 |
| Rural v urban | 0.48 | 0.13 | <0.001 |
| Male v female | 0.25 | 0.15 | 0.090 |
| Year of graduation |  |  | 0.002 |
| 2010-2019 | 0 |  |  |
| 2000-2009 | 0.57 | 0.18 |  |
| 1990-1999 | 0.54 | 0.19 |  |
| <1989 | 0.60 | 0.18 |  |
| Australian v overseas graduate | -0.09 | 0.18 | 0.598 |
| Developing country v other | 0.36 | 0.16 | 0.023 |
|  |  |  |  |
| 2b: Management  ‘Manage common zoonotic diseases’ | | | |
| Veterinarian v GP | 2.71 | 0.15 | <0.001 |
| Rural v urban | 0.51 | 0.13 | <0.001 |
| Male v female | 0.37 | 0.15 | 0.011 |
| Year of graduation |  |  | <0.001 |
| 2010-2019 | 0 |  |  |
| 2000-2009 | 0.56 | 0.18 |  |
| 1990-1999 | 0.48 | 0.19 |  |
| <1989 | 0.73 | 0.18 |  |
| Australian v overseas graduate | -0.14 | 0.18 | 0.43 |
| Developing country v other | 0.26 | 0.15 | 0.087 |
| 2c: Advice  ‘Give advice about prevention of common zoonotic diseases’ | | | |
| Veterinarian v GP | 2.56 | 0.15 | <0.001 |
| Rural v urban | 0.46 | 0.12 | <0.001 |
| Male v female | 0.35 | 0.14 | 0.014 |
| Year of graduation |  |  | 0.019 |
| 2010-2019 | 0 |  |  |
| 2000-2009 | 0.38 | 0.18 |  |
| 1990-1999 | 0.26 | 0.19 |  |
| <1989 | 0.54 | 0.18 |  |
| Australian v overseas graduate | 0.08 | 0.18 | 0.662 |
| Developing country v other | 0.48 | 0.15 | 0.002 |
|  | | | |
| Model 3: Practices  3a: Zoonotic Risk  ‘How often do you discuss potential risk of zoonotic diseases or strategies to prevent these with your patients/clients? ‘ | | | |
| Veterinarian v GP | 0.16 | 0.13 | <0.001 |
| Rural v urban | 0.52 | 0.12 | <0.001 |
| Male v female | -0.01 | 0.14 | 0.933 |
| Year of graduation |  |  | 0.297 |
| 2010-2019 | 0 |  |  |
| 2000-2009 | 0.19 | 0.17 |  |
| 1990-1999 | 0.23 | 0.18 |  |
| <1989 | 0.32 | 0.17 |  |
| Australian v overseas graduate | 0.41 | 0.17 | 0.018 |
| Developing country v other | 0.53 | 0.15 | <0.001 |
|  |  |  |  |
| 3b: Referral  ‘Have you ever recommended a patient with or at risk of a zoonotic disease seek veterinary treatment/management of their animal(s)? / Have you ever recommended a client who has an animal with or at risk of a zoonotic disease seek advice from a GP?’ | | | |
| Veterinarian v GP | 2.89 | 0.17 | <0.001 |
| Rural v urban | -0.34 | 0.16 | 0.027 |
| Male v female | 0.06 | 0.18 | 0.468 |
| Year of graduation |  |  | 0.016 |
| 2010-2019 | 0 |  |  |
| 2000-2009 | 0.69 | 0.23 |  |
| 1990-1999 | 0.48 | 0.24 |  |
| <1989 | 0.42 | 0.22 |  |
| Australian v overseas graduate | 0.12 | 0.21 | 0.81 |
| Developing country v other | 0.45 | 0.19 | 0.019 |

**Table S6 Comparison of mixed practice, equine, exotics and wildlife veterinarians to small animal veterinarians in multivariable logistic regression models for experience, concern, confidence and practices related to zoonoses**

| Explanatory Variable | *b* | SE | p |
| --- | --- | --- | --- |
| Experience with zoonotic diseases  ‘Have you ever diagnosed a zoonotic disease in a patient?’ | | | |
| Other^†^ v Small Animal | -0.69 | 0.41 | 0.027 |
| Rural v urban | 0.05 | 0.40 | 0.957 |
| Male v female | -0.20 | 0.41 | 0.837 |
| Year of graduation |  |  | 0.067 |
| 2010-2019 | 0 |  |  |
| 2000-2009 | 0.60 | 0.43 |  |
| 1990-1999 | 1.01 | 0.56 |  |
| <1989 | 0.85 | 0.48 |  |
| Australian v overseas graduate | -1.26 | 1.03 | 0.105 |
| Developing country v other | 0.65 | 0.55 | 0.204 |
|  | | | |
| Model 1: Concern  ‘How concerned are you about zoonotic diseases in the practice you currently work in?’ | | | |
| Other v Small Animal | 1.12 | 0.21 | <0.001 |
| Rural v urban | 0.30 | 0.20 | 0.135 |
| Male v female | -0.04 | 0.21 | 0.839 |
| Year of graduation |  |  | 0.070 |
| 2010-2019 | 0 |  |  |
| 2000-2009 | -0.09 | 0.23 |  |
| 1990-1999 | 0.53 | 0.24 |  |
| <1989 | 0.12 | 0.24 |  |
| Australian v overseas graduate | 0.56 | 0.30 | 0.059 |
| Developing country v other | 0.40 | 0.22 | 0.067 |
|  |  |  |  |
| Model 2: Confidence  ‘Overall, how confident do you feel in your ability to:’ | | | |
| 2a: Diagnosis  ‘Diagnose common zoonotic diseases’ | | | |
| Other v Small Animal | 0.13 | 0.20 | 0.495 |
| Rural v urban | 0.30 | 0.18 | 0.099 |
| Male v female | -0.14 | 0.20 | 0.467 |
| Year of graduation |  |  | 0.002 |
| 2010-2019 | 0 |  |  |
| 2000-2009 | 0.59 | 0.21 |  |
| 1990-1999 | 0.68 | 0.22 |  |
| <1989 | 0.65 | 0.22 |  |
| Australian v overseas graduate | -0.17 | 0.26 | 0.510 |
| Developing country v other | 0.25 | 0.21 | 0.228 |
|  |  |  |  |
| 2b: Management  ‘Manage common zoonotic diseases’ | | | |
| Other v Small Animal | 0.24 | 0.20 | 0.228 |
| Rural v urban | 0.23 | 0.18 | 0.209 |
| Male v female | -0.13 | 0.20 | 0.528 |
| Year of graduation |  |  | <0.001 |
| 2010-2019 | 0 |  |  |
| 2000-2009 | 0.56 | 0.21 |  |
| 1990-1999 | 0.65 | 0.22 |  |
| <1989 | 0.90 | 0.23 |  |
| Australian v overseas graduate | -0.19 | 0.26 | 0.464 |
| Developing country v other | 0.08 | 0.21 | 0.686 |
| 2c: Advice  ‘Give advice about prevention of common zoonotic diseases’ | | | |
| Other v Small Animal | 0.31 | 0.20 | 0.122 |
| Rural v urban | 0.30 | 0.18 | 0.098 |
| Male v female | 0.05 | 0.20 | 0.817 |
| Year of graduation |  |  | 0.028 |
| 2010-2019 | 0 |  |  |
| 2000-2009 | 0.41 | 0.21 |  |
| 1990-1999 | 0.42 | 0.22 |  |
| <1989 | 0.61 | 0.23 |  |
| Australian v overseas graduate | 0.15 | 0.26 | 0.561 |
| Developing country v other | 0.27 | 0.21 | 0.211 |
|  | | | |
| Model 3: Practices  3a: Zoonotic Risk  ‘How often do you discuss potential risk of zoonotic diseases or strategies to prevent these with your patients/clients? ‘ | | | |
| Other v Small Animal | 0.69 | 0.20 | <0.001 |
| Rural v urban | 0.19 | 0.18 | 0.286 |
| Male v female | 0.12 | 0.20 | 0.922 |
| Year of graduation |  |  | 0.753 |
| 2010-2019 | 0 |  |  |
| 2000-2009 | 0.20 | 0.20 |  |
| 1990-1999 | 0.14 | 0.22 |  |
| <1989 | 0.17 | 0.22 |  |
| Australian v overseas graduate | 0.35 | 0.26 | 0.176 |
| Developing country v other | 0.33 | 0.21 | 0.116 |
|  |  |  |  |
| 3b: Referral  ‘Have you ever recommended a patient with or at risk of a zoonotic disease seek veterinary treatment/management of their animal(s)? / Have you ever recommended a client who has an animal with or at risk of a zoonotic disease seek advice from a GP?’ | | | |
| Other v Small Animal | -0.69 | 0.41 | <0.001 |
| Rural v urban | 0.05 | 0.40 | 0.912 |
| Male v female | -0.20 | 0.41 | 0.705 |
| Year of graduation |  |  | <0.001 |
| 2010-2019 | 0 |  |  |
| 2000-2009 | 0.60 | 0.43 |  |
| 1990-1999 | 1.01 | 0.56 |  |
| <1989 | 0.85 | 0.48 |  |
| Australian v overseas graduate | -1.26 | 1.03 | 0.606 |
| Developing country v other | 0.65 | 0.55 | 0.103 |

**^†^** ‘Other’ includes mixed practice, equine, avian, exotics and wildlife practitioners.
